# IGF2BP3 Overexpression Accelerates Osteoarthritis Progression by Promoting Glycolysis via m^6^A‐Dependent Stabilization of ECM1 mRNA

**DOI:** 10.1002/kjm2.70280

**Published:** 2026-08-19

**Authors:** Xing‐Shi Yuan, Hao‐Jie Zhang, Jia‐Xiang Ding, Zheng‐Liang Luo, Min Chen, Xiao‐Qi Zhang, Xi‐Fu Shang, Zhe Ni, Ke‐Rong Wu

**Affiliations:** ^1^ Department of Orthopedics The First Affiliated Hospital of USTC, Division of Life Sciences and Medicine, University of Science and Technology of China Hefei Anhui China

**Keywords:** ECM1, glycolysis, IGF2BP3, N^6^‐methyladenosine methylation, osteoarthritis

## Abstract

N^6^‐methyladenosine (m^6^A) plays a critical role in osteoarthritis (OA) pathogenesis. This study investigates the role of the m^6^A reader insulin‐like growth factor 2 mRNA‐binding protein 3 (IGF2BP3) in OA progression. OA models were established in vivo through destabilization of the medial meniscus (DMM) surgery in mice and in vitro by stimulating primary chondrocytes with interleukin‐1β (IL‐1β). Cartilage destruction was assessed histopathologically. Chondrocyte viability, proliferation, and apoptosis were measured by CCK‐8, EdU, and flow cytometry, respectively. Inflammatory cytokine levels, lactate, glucose, and total m^6^A were quantified using commercial kits. Expression levels of IGF2BP3, extracellular matrix protein 1 (ECM1), ECM turnover markers, and glycolysis‐related genes were analyzed by RT‐qPCR, Western blot, immunohistochemistry, and immunofluorescence. Potential m^6^A modification sites in ECM1 transcripts were predicted using the SRAMP database, and IGF2BP3‐mediated, m^6^A‐dependent regulation of ECM1 was validated through RNA immunoprecipitation (RIP), MeRIP‐qPCR, and actinomycin D decay assays. Results revealed elevated levels of IGF2BP3, ECM1, m^6^A modification, and glycolytic activity in OA cartilage and IL‐1β‐treated chondrocytes. IGF2BP3 knockdown reversed IL‐1β‐induced reductions in chondrocyte viability and proliferation, suppressed apoptosis, decreased inflammatory cytokine secretion, attenuated ECM degradation, and reduced glycolytic flux in vitro, while also mitigating cartilage damage and matrix breakdown in vivo. Mechanistically, IGF2BP3 stabilized ECM1 mRNA in an m^6^A‐dependent manner, thereby modulating ECM1 expression. Restoration of ECM1 expression abolished the chondroprotective effects of IGF2BP3 knockdown against injury, inflammation, and enhanced glycolysis. In conclusion, IGF2BP3 enhances ECM1 expression via an m^6^A‐dependent pathway, thereby promoting glycolysis and exacerbating OA progression.

## 1 Introduction

Osteoarthritis (OA) represents a widespread degenerative joint condition defined by cartilage matrix depletion, synovial inflammation, and meniscal pathology [1]. Its prevalence increases with age, commonly presenting as joint pain, swelling, and stiffness in middle‐aged and elderly individuals, and may eventually lead to severe mobility impairment [2]. Such symptoms impose considerable physical and emotional strain on those affected. Current treatments for early‐to‐moderate OA mainly rely on analgesics and nonsteroidal anti‐inflammatory drugs, while advanced disease frequently requires joint replacement surgery [3, 4]. Accumulating evidence implicates metabolic pathway dysregulation as a significant contributor to OA advancement [5]. Under stressful environmental conditions, chondrocytes display a metabolic shift away from oxidative phosphorylation toward enhanced glycolysis [6]; consequently, targeting glycolytic pathways might offer a promising avenue for influencing OA progression.

N^6^‐methyladenosine (m^6^A) constitutes a pervasive RNA modification in eukaryotes, affecting nearly all stages of RNA metabolism, including transcription, processing, translation, degradation, and stability control [7, 8]. Within mammalian systems, m^6^A alterations can modulate disease trajectories by affecting mRNA stability. Recent investigations have pinpointed irregular m^6^A patterns in OA. For instance, METTL3‐catalyzed m^6^A methylation of ATG7 promotes autophagic activity in chondrocytes, thereby hastening OA development [9]. Similarly, WTAP‐mediated m^6^A modification of FRZB can instigate inflammatory responses in OA [10]. Furthermore, research underscores the extensive engagement of m^6^A in cancer glycolysis, influencing diverse cellular processes such as chemoresistance, immune escape, and metabolic reprogramming [11, 12]. Nevertheless, whether m^6^A methylation governs aberrant glucose handling in OA remains ambiguous. Insulin‐like growth factor 2 mRNA‐binding protein 3 (IGF2BP3), an m^6^A reader, recognizes m^6^A marks and has been implicated in both tumorigenesis and inflammatory diseases [13, 14]. Recent findings indicate that elevated IGF2BP3 augments glycolytic capacity in hepatocellular carcinoma, heightening tumor drug resistance [15]. Moreover, research by Geng and colleagues suggested that IGF2BP3‐directed m^6^A modification exacerbates articular destruction in rheumatoid arthritis [16]. However, the potential involvement of IGF2BP3 in governing glycolytic processes in OA via m^6^A methylation necessitates further exploration.

Extracellular matrix protein 1 (ECM1) is a secreted glycoprotein contributing to tissue homeostasis maintenance and has been linked to the pathogenesis of various conditions, encompassing liver fibrosis, tumor progression, nephropathy, and inflammatory bowel disease [17, 18, 19, 20]. Importantly, ECM1 is present in osseous and cartilaginous tissues, where it influences chondrocyte proliferation and maturation [21]. Reports suggest a correlation between elevated ECM1 concentrations and heightened OA susceptibility, potentially positioning it as a therapeutic target for this disease [22]. Concurrently, one investigation demonstrated that suppressing ECM1 effectively mitigates OA progression [23]. Despite these observations, the mechanistic function of ECM1 within OA remains incompletely understood. The current study aims to elucidate the prospective mechanism whereby IGF2BP3 regulates OA advancement using both in vivo and in vitro experimental paradigms, thereby furnishing novel perspectives on the fundamental pathogenic processes underlying OA.

## 2 Materials and Methods

### 2.1 Animal Experimentation

Forty male C57BL/6 mice, aged between 10 and 12 weeks, were acquired from Changzhou Kavins Experimental Animal (Changzhou, China). The animals were housed under specific pathogen‐free conditions with an ambient temperature of 23°C–26°C, relative humidity of 50%–70%, and a 12‐h light/dark cycle. Throughout the investigation, all mice had free access to standard chow and autoclaved water. All animal procedures were reviewed and approved by the Animal Ethics Committee of the First Affiliated Hospital of USTC (2021‐N(H)‐110). Mice were randomly assigned to four experimental cohorts (*n* = 10 per group): Sham, OA, AAV‐NC, and AAV‐sh‐IGF2BP3. An OA model was induced in all groups except the Sham group via destabilization of the medial meniscus (DMM) surgery, performed as previously described [24]. Briefly, following intraperitoneal anesthesia using 1% pentobarbital sodium (50 mg/kg), the medial meniscotibial ligament of the right knee joint was severed. In Sham‐operated animals, the joint capsule was opened to expose the knee, leaving the ligament untouched. One week prior to surgery, intra‐articular injections were administered into the right knee joint. Mice belonging to the AAV‐NC and AAV‐sh‐IGF2BP3 groups received 10 μL of solution containing adeno‐associated virus (AAV) carrying either a negative control sequence or a short hairpin RNA targeting IGF2BP3 (Sangon, Shanghai, China), at a concentration of 1 × 10^11^ vg/mL. Eight weeks postsurgery, all animals were euthanized via cervical dislocation. Subsequently, cartilage tissues from the knee joints were collected. One portion was snap‐frozen at −80°C for subsequent Western blot, PCR, and ELISA analyses, while the other portion was fixed, decalcified, and embedded in paraffin for histopathological examination.

### 2.2 Histopathological Analysis

Knee joint specimens were harvested immediately following euthanasia and immersed in 4% paraformaldehyde for 48 h. Thereafter, decalcification was performed employing 10% ethylene diamine tetraacetic acid (EDTA) over a 4‐week duration. Following decalcification, tissues underwent graduated dehydration through an ascending ethanol series (75%, 85%, 90%, and 95%), were exposed to absolute ethanol for 2 h, and subsequently cleared in xylene for 10 min. After these procedures, the samples were embedded in paraffin and sectioned at 5 μm thickness. Paraffin sections were deparaffinized using xylene and rehydrated through a graded ethanol series prior to staining. To evaluate general morphology, hematoxylin–eosin (H&E) staining (Sigma‐Aldrich, St. Louis, Missouri, USA) was performed, with hematoxylin applied for 5 min followed by eosin for 40 s. For assessing cartilage matrix composition, safranin O‐fast green staining (Sangon) was conducted. This involved sequential immersion in fast green solution (1 min), acetic acid solution (30 s), and safranin O solution (15 min). Poststaining, all sections were dehydrated, cleared, and mounted for microscopic observation. Histopathological changes were visualized and documented under a light microscope. The extent of articular cartilage destruction in OA mice was quantified according to the Osteoarthritis Research Society International (OARSI) scoring system [25].

### 2.3 Immunohistochemistry

Following deparaffinization in xylene and rehydration through a graded ethanol series, paraffin‐embedded knee joint sections (5 μm thickness) were processed for immunohistochemical analysis. Antigen retrieval was achieved by heating the sections in sodium citrate buffer for 10 min. Endogenous peroxidase activity was quenched by incubation with 3% hydrogen peroxide for 20 min at room temperature, after which nonspecific binding sites were blocked with 5% goat serum for 30 min. After rinsing with phosphate‐buffered saline (PBS), the sections were incubated overnight at 4°C with primary antibodies against IGF2BP3 (14642‐1‐AP, 1:300 dilution; Proteintech, Rosemont, Illinois, USA) or ECM1 (ab126629, 1:200 dilution; Abcam, Cambridge, UK). The following day, the sections were incubated with a horseradish peroxidase‐conjugated secondary antibody (ab205718, 1:3000 dilution; Abcam) for 30 min at room temperature. Immunoreactivity was visualized by sequential application of 3,3′‐Diaminobenzidine chromogen and hematoxylin counterstaining, followed by dehydration and clearing. Stained sections were examined under a light microscope, and images were captured for analysis. The positively stained areas corresponding to each target protein were quantified using ImageJ software.

### 2.4 Primary Chondrocyte Culture and Treatment

Primary murine chondrocytes were isolated following an established protocol [26]. Briefly, cartilage tissues harvested from mouse knee joints under aseptic conditions were finely minced and then digested sequentially with 0.25% trypsin for 30 min and 0.2% collagenase for 6 h at 37°C. The resulting cell suspension was filtered through a 70 μm cell strainer and centrifuged at 1000 rpm for 5 min to collect the chondrocytes. Isolated cells were maintained in DMEM medium (Procell, Wuhan, China) supplemented with 10% fetal bovine serum (Excell, Shanghai, China) and 1% penicillin–streptomycin (Biosharp, Hefei, China), and cultured at 37°C in a humidified environment containing 5% CO_2_. To establish an in vitro OA model, chondrocytes were stimulated with 10 ng/mL interleukin‐1β (IL‐1β) for 24 h [27]. For specific experiments, cells were treated with the selective m^6^A methylation inhibitor 3‐Deazaadenosine (DAA, 50 μM; MedChemExpress, Shanghai, China) for 24 h.

### 2.5 Cell Transfection

Small interfering RNAs (siRNAs) targeting IGF2BP3 and METTL3 (si‐IGF2BP3, si‐METTL3), along with their negative control (si‐NC), were sourced from Sangon. Additionally, plasmids for overexpressing IGF2BP3 and ECM1 (ov‐IGF2BP3, ov‐ECM1), along with the empty vector control (ov‐NC), were obtained from the same manufacturer. For transfection, chondrocytes were seeded into 24‐well culture plates and cultured until reaching approximately 80% confluence. Subsequently, siRNA constructs or plasmid DNA were introduced into the cells employing Lipofectamine 3000 reagent (Invitrogen, Carlsbad, California, USA). At 48 h posttransfection, the efficiency of gene knockdown or overexpression was verified by real‐time quantitative polymerase chain reaction (RT‐qPCR) and Western blot analysis.

### 2.6 Western Blot

Total protein was extracted from harvested mouse cartilage tissues or cultured chondrocytes utilizing RIPA lysis buffer (Biosharp). Following centrifugation, the supernatant containing total protein was collected. Protein samples of equal quantity were then separated by SDS‐PAGE and electrophoretically transferred onto PVDF membranes. Nonspecific binding was blocked by incubating the membranes in 5% skim milk at room temperature. Membranes were then incubated overnight at 4°C with primary antibodies, followed by incubation with appropriate horseradish peroxidase‐conjugated secondary antibodies (goat anti‐rabbit or anti‐mouse; 1:2000 dilution; ab6721, ab205719, Abcam) for 1 h at ambient temperature. After three washes with TBST, protein signals were visualized utilizing an ECL ultra‐sensitive chemiluminescence detection kit (Epizyme, Shanghai, China) and imaged with a chemiluminescence imaging system (Bio‐Rad, Hercules, California, USA). Band intensities were quantified employing ImageJ software to assess relative protein expression levels. The primary antibodies used in this study were as follows: IGF2BP3 (1:20000, Cat. No. 14642‐1‐AP, Proteintech), ECM1 (1:1000, Cat. No. ab126629, Abcam), MMP‐13 (1:1000, Cat. No. #69926, Cell Signaling Technology, Danvers, Massachusetts, USA), ADAMTS5 (1:250, Cat. No. ab41037, Abcam), collagen II (1:1000, Cat. No. ab307674, Abcam), aggrecan (1:1000, Cat. No. ab3778, Abcam), and GAPDH (1:4000, Cat. No. 10494‐1‐AP, Proteintech).

### 2.7 RT‐qPCR

Total RNA was extracted from articular cartilage tissues and cultured chondrocytes utilizing TRIzol reagent (Takara, Shiga, Japan), adhering to the manufacturer's protocol. Reverse transcription was carried out with the PrimeScript RT Reagent Kit (Takara) to synthesize complementary DNA (cDNA). Quantitative real‐time PCR (qPCR) was subsequently performed on an ABI 7500 Fast Real‐Time PCR system using SYBR Green PCR Master Mix (Solarbio, Beijing, China). GAPDH served as the internal reference gene for normalization. Relative quantification of target gene mRNA expression was calculated employing the 2^−ΔΔCt^ method. The specific primer sequences used for amplification are provided in Table 1.

**TABLE 1:** Primer sequences for RT‐qPCR.

Gene	Forward primer (5′ → 3′)	Reverse primer (5′ → 3′)
IGF2BP3	5′‐GTTGGGGAGGAAGAGACAAAAAGTA‐3′	5′‐TCCAGCACCTCCCATTGTAA‐3′
ECM1	5′‐GCTGTCCCTCAAGAAGCCAT‐3′	5′‐GGTCATTGCATCCTCCCACA‐3′
GLUT1	5′‐GCGGGAGACGCATAGTTACA‐3′	5′‐ACCTTAGGCCCTGATTCCTCT‐3′
LDHA	5′‐GGAGCAACTTGGCGCTCTAC‐3′	5′‐GTTTCGCTGGACCAGGTTGA‐3′
HK2	5′‐CGCCGGATTGGAACAGAACT‐3′	5′‐CTGTCACCCTTACTCGGAGC‐3′
PKM2	5′‐GTCCGCTCTAGGTATCGCAG‐3′	5′‐CACCATGTCCACATCCTGCT‐3′
GAPDH	5′‐AGAGGGATGCTGCCCTTACC‐3′	5′‐TCGTGGTTCACACCCATCAC‐3′

### 2.8 Cell Proliferation Assessment

Cell proliferation was determined with the Cell Counting Kit‐8 (CCK‐8) and 5‐ethynyl‐2′‐deoxyuridine (EdU) incorporation assays. For the CCK‐8 assay, chondrocytes were plated in 96‐well plates at a density of 5 × 10^3^ cells per well and cultured for 24 h at 37°C. Subsequently, 10 μL of CCK‐8 reagent (Biosharp) was added to each well, followed by a 2‐h incubation. Absorbance at 450 nm was measured with a microplate reader. The EdU assay was performed employing the Cell‐Light EdU Apollo In Vitro Kit (Ribobio, Guangzhou, China). Following the manufacturer's protocol, cells were incubated with EdU labeling solution for 2 h. After PBS washing, cells were fixed with 4% paraformaldehyde and permeabilized with 0.5% Triton X‐100. Apollo staining solution was employed to visualize proliferating cells, and nuclei were counterstained with 4′,6‐diamidino‐2‐phenylindole (DAPI). Images were acquired using a fluorescence microscope (Olympus, Tokyo, Japan), and the percentage of EdU‐positive cells was calculated with ImageJ software.

### 2.9 Apoptosis Assessment

Flow cytometry was utilized to evaluate chondrocyte apoptosis employing an Annexin V‐FITC/PI Apoptosis Detection Kit (Bestbio, Shanghai, China). Briefly, chondrocytes were detached using 0.25% trypsin, and the resulting cell suspension was combined with washing buffer followed by centrifugation at 300×*g* for 5 min. Cells were rinsed twice with chilled PBS and then resuspended in 400 μL of Annexin V binding buffer. Subsequently, 5 μL of Annexin V‐FITC staining solution was added to the suspension, gently agitated, and incubated at 4°C protected from light for 15 min. Following this, 10 μL of PI staining solution was introduced, mixed gently, and incubated under the same conditions for an additional 5 min. Samples were analyzed immediately using a flow cytometer.

### 2.10 Immunofluorescence

Immunofluorescence staining was performed to evaluate protein expression. Chondrocytes were seeded in 12‐well culture plates at a density of 1 × 10^5^ cells per well. Following phosphate‐buffered saline (PBS) rinsing, cells were fixed with 4% paraformaldehyde for 10 min at ambient temperature and subsequently blocked with 5% bovine serum albumin for 1 h. Cells were then incubated overnight at 4°C with primary antibodies. Antibodies utilized were specific for IGF2BP3 (dilution 1:100, 14642‐1‐AP, Proteintech) and ECM1 (dilution 1:200, ab126629, Abcam). After washing, cells were incubated with a fluorescent secondary antibody (goat anti‐rabbit, 1:500 dilution, ab150077, Abcam) for 1 h at room temperature. Nuclei were stained with 4′,6‐diamidino‐2‐phenylindole (DAPI). Fluorescent images were captured employing a fluorescence microscope (Olympus).

### 2.11 Enzyme‐Linked Immunosorbent Assay (ELISA)

Concentrations of the pro‐inflammatory cytokines IL‐6, TNF‐α (Elabscience, Wuhan, China), and IL‐8 (R&D Systems, Abingdon, UK) were quantified in both mouse cartilage tissue homogenates and chondrocyte culture supernatants. Commercially sourced ELISA kits (Beyotime, Shanghai, China) were utilized for this purpose, with all procedures strictly adhering to the protocols provided by the manufacturer.

### 2.12 Lactate and Glucose Level Detection

Lactate and glucose levels were quantified using dedicated assay kits (Lactate Assay Kit and Glucose Assay Kit, respectively; Sigma‐Aldrich), in accordance with the manufacturer's instructions. Chondrocytes were homogenized on ice using the appropriate buffer—either Glucose Assay Buffer or Lactate Assay Buffer. Following homogenization, supernatants were collected via centrifugation at 3280×*g* for 5 min at 25°C. To eliminate protein interference, samples were processed employing ultrafiltration devices. For glucose determination, prepared samples were diluted with Glucose Assay Buffer, and fluorescence intensity was subsequently measured (excitation: 535 nm, emission: 587 nm). For lactate measurement, samples were diluted with Lactate Assay Buffer, and absorbance was read at 450 nm.

### 2.13 m^6^A RNA Methylation Quantification

Quantification of m^6^A modifications in mRNA was conducted employing an m^6^A Methylation Quantification Kit (A&D Technology, Beijing, China). According to the manufacturer's instructions, total RNA extracted from cartilage tissues or cultured chondrocytes was subjected to colorimetric detection of m^6^A levels. In brief, mRNA samples were initially incubated with 80 μL of Binding Solution at 37°C for 90 min, followed by 50 μL of Capture Solution at room temperature for another 90 min. This step was sequentially followed by the addition of 50 μL of Detection Solution (incubated for 30 min at room temperature) and then 50 μL of diluted Enhancer Solution (incubated for an additional 30 min at room temperature). Following washes with Wash Buffer, each well received 100 μL of Developer Solution and was incubated for 5 min at room temperature protected from light. Absorbance was subsequently measured at 450 nm using a microplate reader.

### 2.14 RNA Immunoprecipitation (RIP) Assay

RIP assays were performed employing the Magna RIP RNA‐Binding Protein Immunoprecipitation Kit (Millipore, Burlington, Massachusetts, USA). For antibody coupling, magnetic beads were incubated with either anti‐IGF2BP3 antibody or a control anti‐IgG antibody for 2 h at 4°C. Chondrocytes were lysed using RIP Lysis Buffer, and the resulting lysate was incubated overnight with the antibody‐bead complexes at 4°C to immunoprecipitate target RNA. Subsequently, RNA bound to the beads was isolated employing TRIzol reagent, and the immunoprecipitated material was retained for downstream analysis. Enrichment of specific transcripts was then quantified by RT‐qPCR using gene‐specific primers.

### 2.15 Methylated RNA Immunoprecipitation‐qPCR (MeRIP‐qPCR)

Total RNA was extracted from chondrocytes or tissue specimens utilizing TRIzol reagent (Takara), adhering to the manufacturer's instructions. Purified mRNA was chemically fragmented into approximately 100‐nucleotide fragments with the Ambion Fragmentation Reagent (Thermo Fisher Scientific, Waltham, Massachusetts, USA). To immunoprecipitate methylated RNA, fragmented mRNA was incubated for 4 h at 4°C with Protein A/G magnetic beads preconjugated with 1 μg of an m^6^A‐specific antibody (ab284130, Abcam). Following RNA elution from the beads, the enrichment of specific methylated transcripts was determined by RT‐qPCR.

### 2.16 Actinomycin D Treatment

To assess mRNA stability, chondrocytes were treated with Actinomycin D at a concentration of 5 μg/mL (Sigma‐Aldrich), added directly to the culture medium. Cells were harvested at designated time points (0, 2, 4, and 6 h) following Actinomycin D addition. Total RNA was subsequently extracted using TRIzol reagent, and the residual levels of ECM1 mRNA were measured by RT‐qPCR. For each sample, ECM1 mRNA abundance was normalized to the value at the 0‐h time point.

### 2.17 Data Analysis

Data were processed and analyzed employing GraphPad Prism 8.0 software (San Diego, California, USA). Results were presented as mean ± standard deviation. Comparisons between two groups were performed using a *t*‐test. Comparisons among multiple groups were performed using one‐way analysis of variance followed by Tukey's post hoc test. A *p*‐value < 0.05 was considered statistically significant.

## 3 Results

### 3.1 m^6^A, IGF2BP3, and ECM1 Levels are Upregulated in OA Cartilage and IL‐1β‐Stimulated Chondrocytes

**FIGURE 1:**
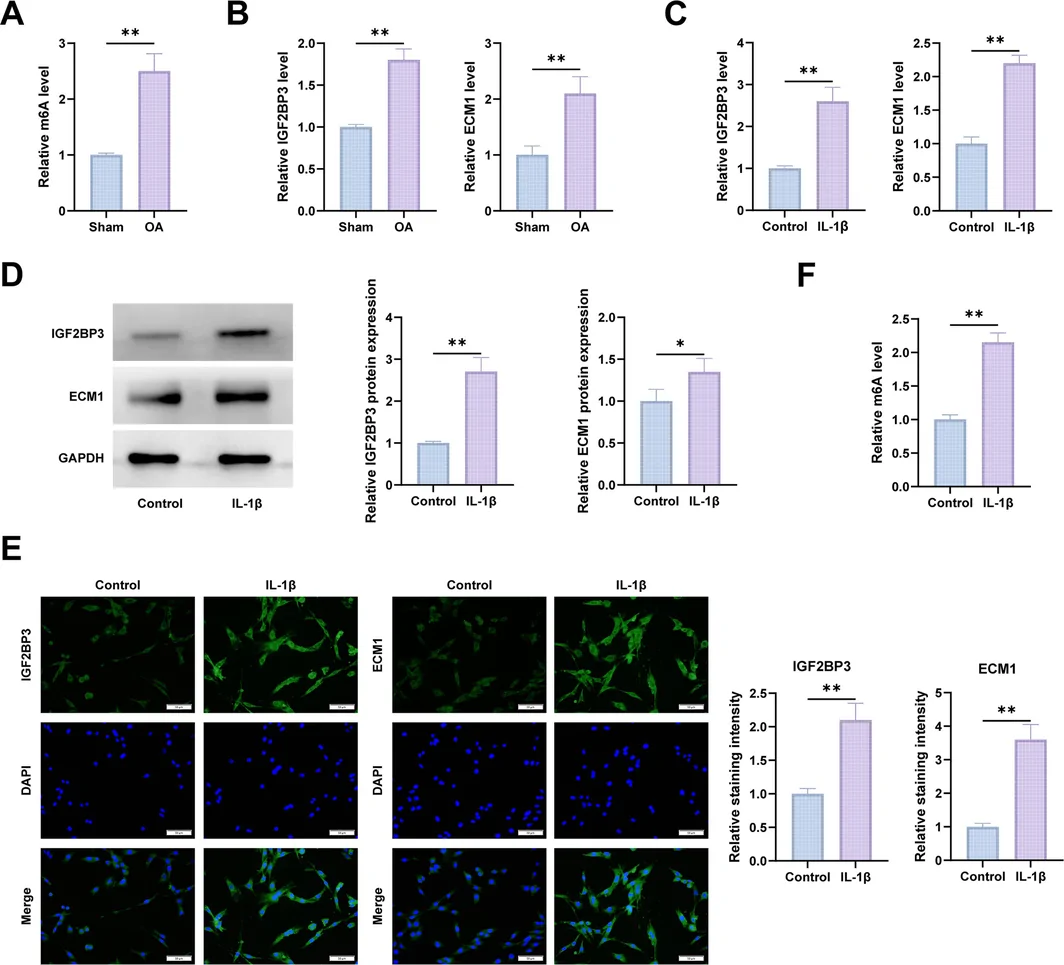
m^6^A, IGF2BP3, and ECM1 levels are upregulated in OA cartilage and IL‐1β‐stimulated chondrocytes. (A) m^6^A levels in mouse cartilage tissue were measured using an m^6^A RNA methylation quantification kit, *n* = 6; (B) RT‐qPCR was used to detect the levels of IGF2BP3 and ECM1 in the mouse model, *n* = 6; (C, D) RT‐qPCR and Western blot were used to detect the mRNA and protein expression levels of IGF2BP3 and ECM1 in chondrocytes, *n* = 3; (E) immunofluorescence was used to observe the expression of IGF2BP3 and ECM1 in chondrocytes, *n* = 3; (F) m^6^A levels in chondrocytes were measured using an m^6^A RNA methylation quantification kit, *n* = 3. Data are presented as mean ± SD. * *p* < 0.05, **p < 0.01.

Aberrant m^6^A modification has been well documented in OA. To investigate its involvement, global m^6^A levels were assessed using a methylation quantification kit. Compared to Sham controls, cartilage tissues from OA mice exhibited significantly elevated m^6^A levels (Figure 1A). Subsequently, the expression of IGF2BP3, a key reader of m^6^A marks, was examined. RT‐qPCR analysis revealed a marked increase in IGF2BP3 mRNA within OA cartilage relative to the Sham group (Figure 1B). Given prior reports linking ECM1 to OA risk, its expression was also evaluated. ECM1 mRNA levels were substantially upregulated in OA cartilage tissues compared to Sham controls (Figure 1B). Consistent with these in vivo observations, an in vitro inflammatory model using IL‐1β‐stimulated chondrocytes demonstrated significant increases in both IGF2BP3 and ECM1 at the mRNA and protein levels (Figure 1C,D). Immunofluorescence staining further corroborated these findings, showing enhanced fluorescence intensity for both proteins following IL‐1β treatment (Figure 1E). Additionally, m^6^A levels were significantly higher in IL‐1β‐stimulated chondrocytes than in untreated controls (Figure 1F). Collectively, these data demonstrate that OA is characterized by elevated global m^6^A levels, alongside increased expression of IGF2BP3 and ECM1.

### 3.2 Effects of IGF2BP3 Expression Levels on IL‐1β‐Induced Chondrocyte Injury

**FIGURE 2:**
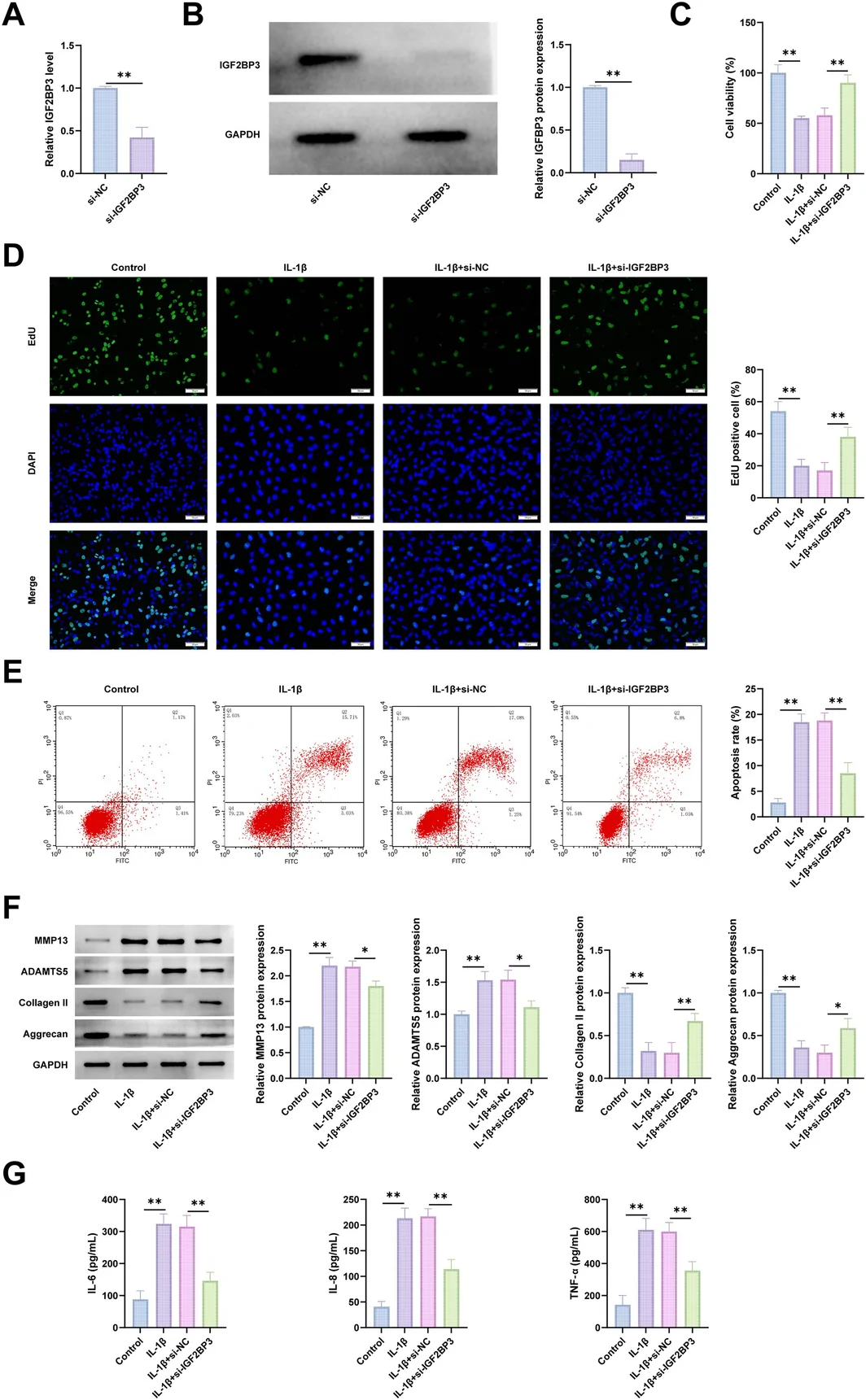
Effects of IGF2BP3 expression levels on IL‐1β‐induced chondrocyte injury. (A, B) RT‐qPCR and Western blot were used to verify the knockdown efficiency of IGF2BP3; (C) CCK‐8 assay was used to detect chondrocyte viability; (D) EdU staining was used to detect chondrocyte proliferation; (E) flow cytometry was used to assess chondrocyte apoptosis; (F) Western blot was used to examine the expression levels of ECM degradation and cartilage synthesis‐related proteins (MMP13, ADAMTS5, collagen II, aggrecan); (G) ELISA was used to detect the levels of pro‐inflammatory cytokines IL‐8, IL‐6, and TNF‐α in the chondrocyte supernatant, *n* = 3. Data are presented as mean ± SD. *p < 0.05, **p < 0.01.

To elucidate the functional role of IGF2BP3 in OA pathogenesis, loss‐of‐function experiments were performed in chondrocytes. Successful knockdown of IGF2BP3 was first validated by RT‐qPCR and Western blot, which confirmed significant reductions in both its mRNA and protein levels (Figure 2A,B). The CCK‐8 assay revealed that exposure to IL‐1β markedly impaired chondrocyte viability; however, this detrimental effect was significantly reversed upon IGF2BP3 knockdown (Figure 2C). EdU incorporation assays demonstrated that the IL‐1β‐induced decrease in proliferative activity was substantially reversed following IGF2BP3 knockdown (Figure 2D). Flow cytometric analysis further indicated that IGF2BP3 downregulation effectively attenuated IL‐1β‐triggered apoptosis in chondrocytes (Figure 2E). Examination of ECM‐related markers showed that IL‐1β stimulation significantly upregulated the degradative enzymes MMP13 and ADAMTS5, while concomitantly suppressing the expression of anabolic components collagen II and aggrecan. Notably, these alterations were effectively counteracted by IGF2BP3 knockdown (Figure 2F). Moreover, ELISA measurements revealed that the secretion of pro‐inflammatory cytokines, including IL‐8, IL‐6, and TNF‐α, was significantly elevated following IL‐1β stimulation. As anticipated, this inflammatory response was markedly attenuated in cells transfected with si‐IGF2BP3 (Figure 2G). Collectively, these results demonstrate that IGF2BP3 knockdown exerts protective effects on IL‐1β‐stimulated chondrocytes by enhancing cell viability and proliferation, suppressing apoptosis, and mitigating ECM degradation.

### 3.3 Effects of IGF2BP3 on Chondrocyte Glycolysis

**FIGURE 3:**
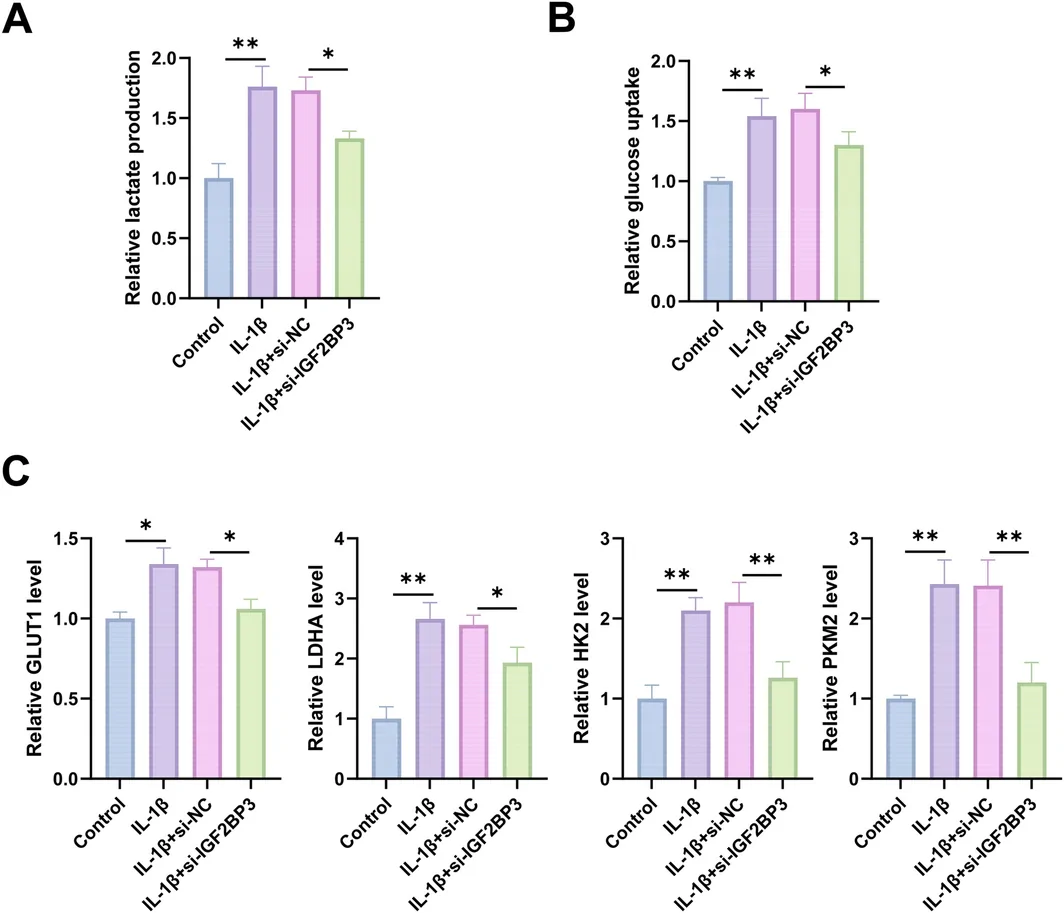
Effects of IGF2BP3 on chondrocyte glycolysis. (A) Measurement of lactate production in chondrocytes; (B) measurement of glucose consumption in chondrocytes; (C) RT‐qPCR was used to detect the mRNA levels of glycolysis‐related genes GLUT1, LDHA, HK2, and PKM2, *n* = 3. Data are presented as mean ± SD. *p < 0.05, **p < 0.01.

Given the established role of glycolysis in OA progression, the potential link between IGF2BP3 expression and glycolytic activity was examined. Chondrocytes stimulated with IL‐1β exhibited a significant increase in both lactate production (Figure 3A) and glucose consumption (Figure 3B) compared to untreated controls. Consistently, RT‐qPCR analysis revealed that the mRNA expression levels of key glycolytic enzymes—including GLUT1, LDHA, HK2, and PKM2—were markedly elevated following IL‐1β treatment (Figure 3C). Conversely, knockdown of IGF2BP3 significantly attenuated these effects. Compared to cells treated with IL‐1β plus negative control siRNA (IL‐1β + si‐NC), those with IGF2BP3 knockdown showed reduced glucose uptake and lower lactate output (Figure 3A,B). In parallel, the upregulation of glycolysis‐related gene transcripts induced by IL‐1β was also reversed upon IGF2BP3 knockdown (Figure 3C). These findings indicate that IGF2BP3 positively regulates the glycolytic pathway in chondrocytes under inflammatory conditions.

### 3.4 IGF2BP3 Recognizes m^6^A Methylation Sites on ECM1 mRNA

**FIGURE 4:**
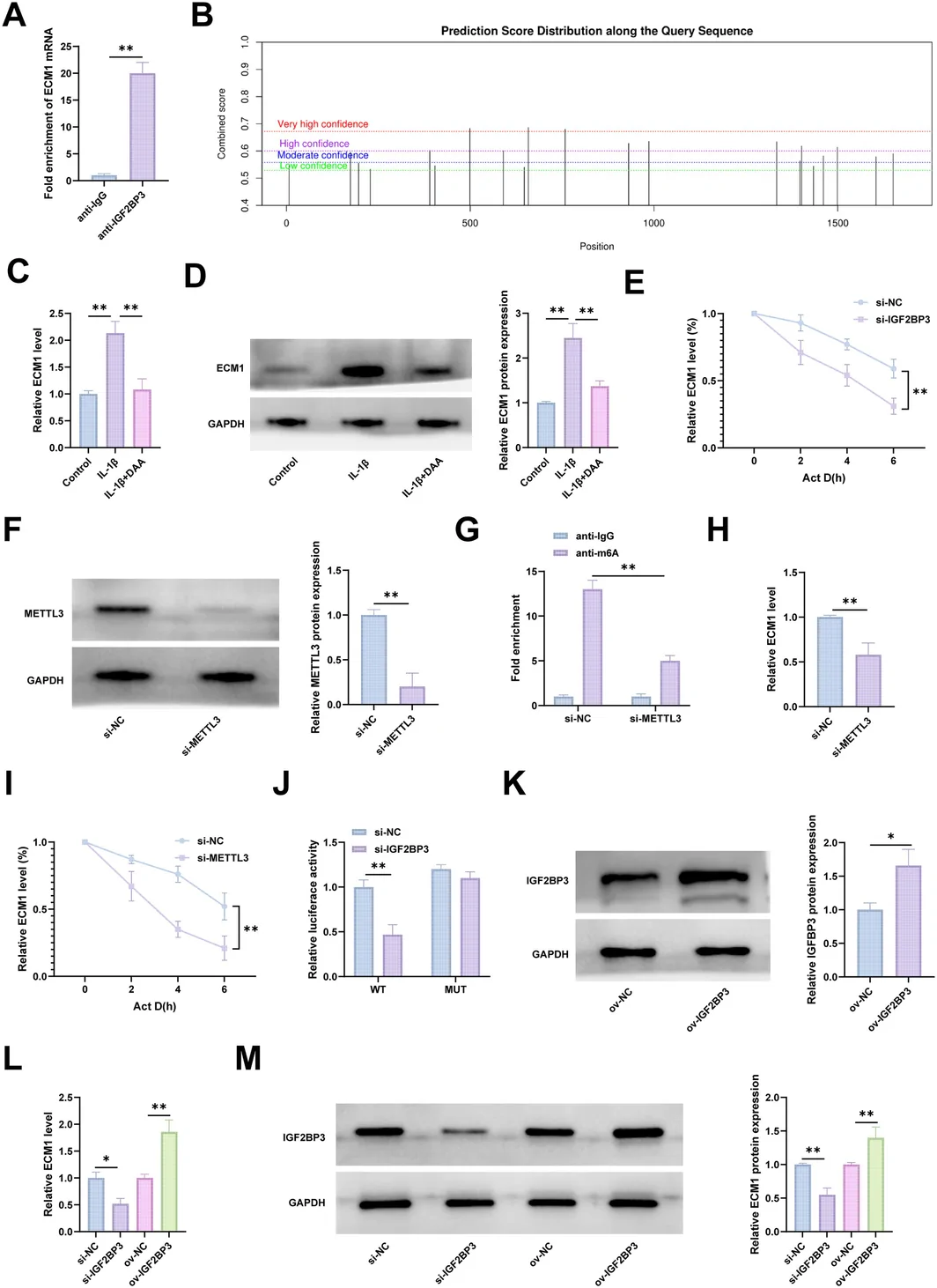
IGF2BP3 stabilizes ECM1 mRNA levels via m^6^A methylation. (A) RIP assay was used to verify the binding of IGF2BP3 to ECM1 mRNA; (B) the SRAMP database was used to predict m^6^A methylation sites on ECM1; (C, D) RT‐qPCR and Western blot were used to detect the effect of an m^6^A methylation inhibitor on ECM1 levels in chondrocytes; (E) Actinomycin D assay was used to evaluate the effect of IGF2BP3 knockdown on the half‐life of ECM1 mRNA; (F) Western blot was used to detect the knockdown efficiency of METTL3; (G) MeRIP‐qPCR was used to analyze the m^6^A modification level of ECM1; (H) RT‐qPCR was used to detect ECM1 mRNA levels; (I) Actinomycin D assay was used to evaluate the effect of METTL3 knockdown on the half‐life of ECM1 mRNA; (J) detection of wild‐type and mutant luciferase reporter gene activity after IGF2BP3 knockdown; (K) Western blot was used to verify the overexpression efficiency of IGF2BP3; (L, M) RT‐qPCR and Western blot were used to detect ECM1 expression levels after IGF2BP3 knockdown or overexpression, *n* = 3. Data are presented as mean ± SD. *p < 0.05, **p < 0.01.

IGF2BP3 is a well‐established m^6^A reader that stabilizes target transcripts in an m^6^A‐dependent manner, thereby modulating gene expression. To investigate whether IGF2BP3 interacts with ECM1 mRNA, RIP assays were conducted. Compared to the control IgG group, ECM1 mRNA was significantly enriched in the anti‐IGF2BP3 pull‐down complex (Figure 4A), indicating a specific association between IGF2BP3 and ECM1 transcripts. Bioinformatic analysis using the SRAMP database further predicted multiple potential m^6^A modification sites within ECM1 mRNA (Figure 4B). Functional experiments demonstrated that pharmacological inhibition of m^6^A methylation with DAA markedly reduced ECM1 expression in IL‐1β‐stimulated chondrocytes (Figure 4C,D). Actinomycin D chase assays revealed that knockdown of IGF2BP3 significantly shortened the half‐life of ECM1 mRNA, implying a role for IGF2BP3 in maintaining ECM1 transcript stability (Figure 4E). To further delineate the involvement of m^6^A modification, METTL3—a key m^6^A methyltransferase—was knocked down in chondrocytes (knockdown efficiency shown in Figure 4F). MeRIP‐qPCR analysis revealed that METTL3 knockdown substantially decreased the m^6^A enrichment on ECM1 mRNA (Figure 4G), accompanied by a reduction in ECM1 mRNA levels (Figure 4H) and accelerated decay of ECM1 transcripts (Figure 4I). These findings underscore the dependence of ECM1 mRNA stability on m^6^A modification. To determine whether IGF2BP3 recognizes specific m^6^A sites within ECM1, luciferase reporter constructs harboring either wild‐type (WT) or mutant m^6^A consensus sequences were generated. In cells transfected with the WT‐ECM1 reporter, IGF2BP3 knockdown led to a significant decrease in luciferase activity; however, this effect was abrogated when the m^6^A sites were mutated (Figure 4J). Consistently, modulation of IGF2BP3 expression in IL‐1β‐treated chondrocytes revealed that IGF2BP3 knockdown downregulated both ECM1 mRNA and protein levels, whereas overexpression of IGF2BP3 upregulated ECM1 expression (Figure 4K–M). Collectively, these data indicate that IGF2BP3 binds to m^6^A‐modified sites on ECM1 mRNA, thereby enhancing its stability and upregulating ECM1 expression under inflammatory conditions.

### 3.5 ECM1 Overexpression Reverses the Ameliorative Effects of IGF2BP3 Knockdown on IL‐1β‐Stimulated Chondrocyte Injury and Glycolysis

**FIGURE 5:**
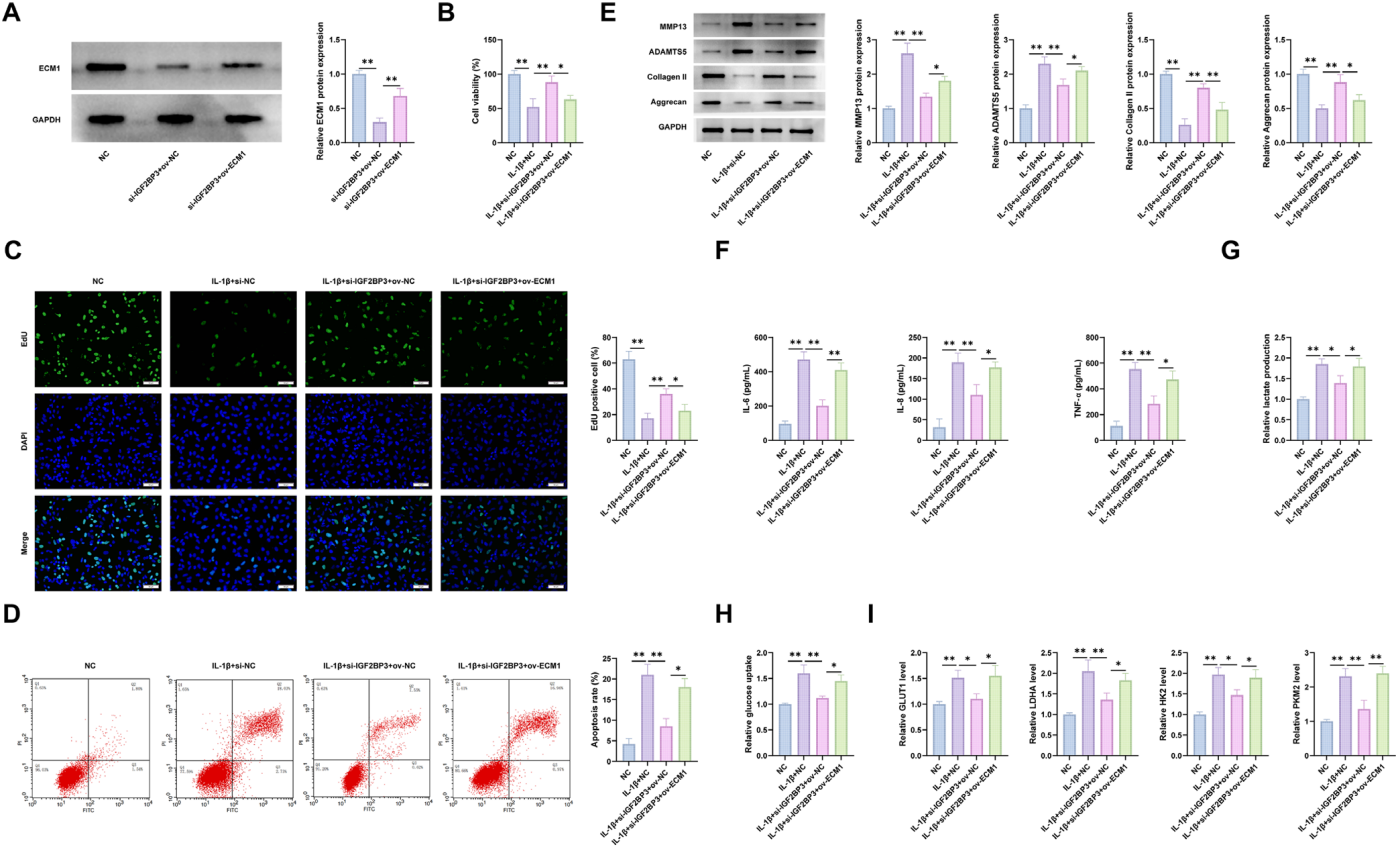
ECM1 overexpression reverses the ameliorative effects of IGF2BP3 knockdown on IL‐1β‐stimulated chondrocyte injury and glycolysis. (A) Western blot was used to detect ECM1 expression levels; (B) CCK‐8 assay was used to detect chondrocyte viability; (C) EdU staining was used to detect chondrocyte proliferation; (D) flow cytometry was used to assess chondrocyte apoptosis; (E) Western blot was used to examine the expression levels of ECM degradation and cartilage synthesis‐related proteins (MMP13, ADAMTS5, collagen II, aggrecan); (F) ELISA was used to detect the levels of pro‐inflammatory cytokines IL‐8, IL‐6, and TNF‐α in the chondrocyte supernatant; (G) measurement of lactate production in chondrocytes; (H) measurement of glucose consumption in chondrocytes; (I) RT‐qPCR was used to detect the mRNA levels of glycolysis‐related genes GLUT1, LDHA, HK2, and PKM2, *n* = 3. Data are presented as mean ± SD. *p < 0.05, **p < 0.01.

To determine whether IGF2BP3 exerts its effects in OA through the regulation of ECM1, rescue experiments were designed in IL‐1β‐stimulated chondrocytes. Cells were transfected with si‐IGF2BP3 alone or together with ov‐ECM1 (a plasmid for ECM1 overexpression). Western blot analysis confirmed that co‐transfection with ov‐ECM1 effectively restored ECM1 protein levels that had been suppressed by IGF2BP3 knockdown (Figure 5A). Functional assays demonstrated that the enhancement of chondrocyte viability mediated by si‐IGF2BP3 under IL‐1β stimulation was significantly diminished upon ECM1 overexpression (Figure 5B). Similarly, EdU incorporation and flow cytometry analyses revealed that the proliferative and anti‐apoptotic effects of IGF2BP3 knockdown were both counteracted by ECM1 overexpression (Figure 5C,D). Examination of cartilage matrix markers indicated that the reductions in MMP13 and ADAMTS5, as well as the increases in collagen II and aggrecan resulting from IGF2BP3 knockdown, were partially reversed by ECM1 overexpression (Figure 5E). Furthermore, ELISA assays showed that the suppressive effect of si‐IGF2BP3 on the secretion of pro‐inflammatory cytokines IL‐8, IL‐6, and TNF‐α was significantly attenuated in the presence of ov‐ECM1 (Figure 5F). Evaluation of glycolytic activity revealed that ECM1 overexpression also reversed the metabolic alterations induced by IGF2BP3 knockdown. Compared to cells transfected with si‐IGF2BP3 alone, those co‐transfected with ov‐ECM1 exhibited significantly increased lactate production and glucose consumption (Figure 5G,H). Consistently, the mRNA levels of glycolysis‐related genes GLUT1, LDHA, HK2, and PKM2 were upregulated following ECM1 overexpression (Figure 5I). Collectively, these findings indicate that IGF2BP3 modulates chondrocyte injury and glycolysis in OA, at least in part, through the regulation of ECM1 expression.

### 3.6 IGF2BP3 Knockdown Significantly Inhibits Glycolytic Activity and Alleviates OA in Mice

**FIGURE 6:**
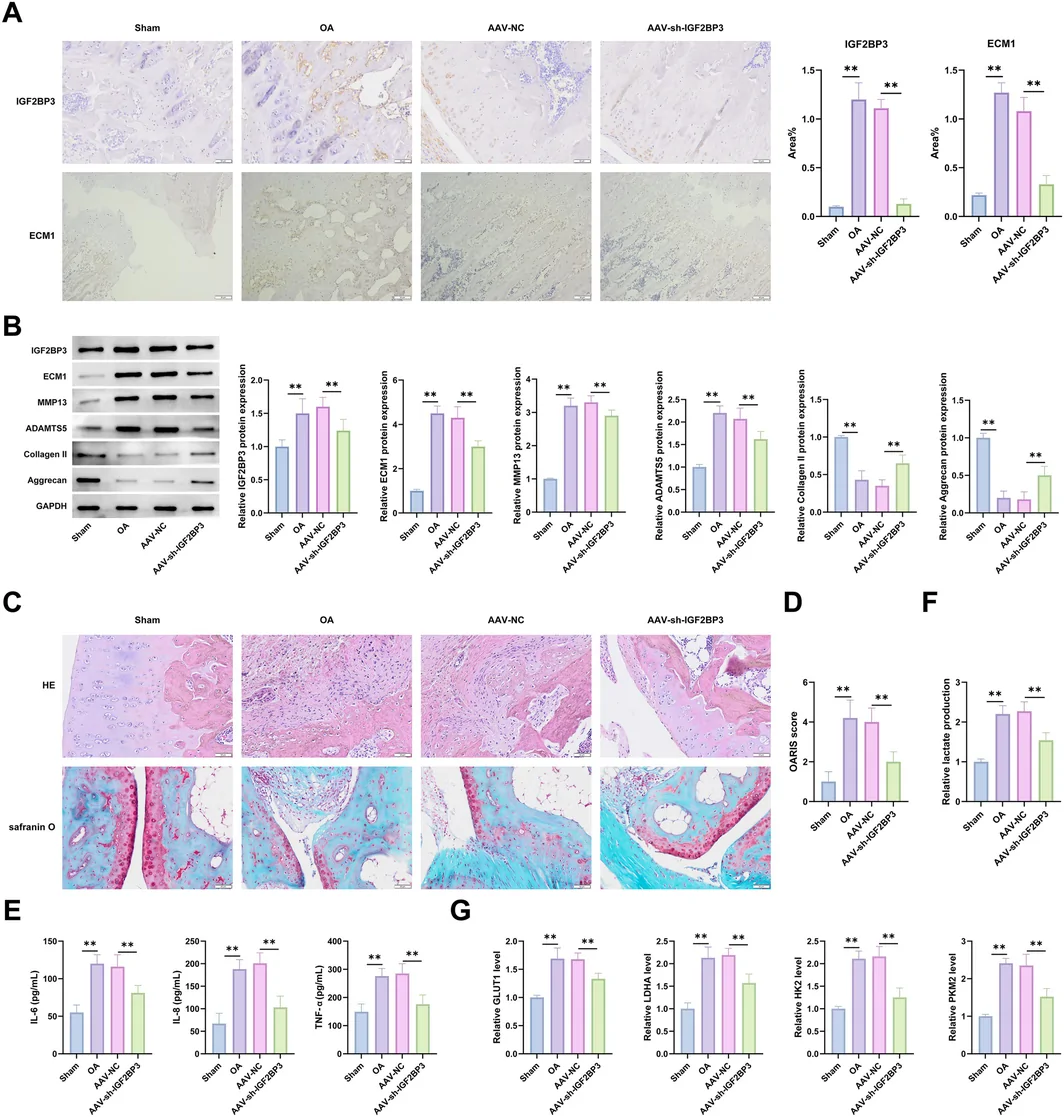
IGF2BP3 knockdown significantly inhibits glycolytic activity and alleviates OA in mice. (A) Immunohistochemistry was used to observe the expression of IGF2BP3 and ECM1 in the knee cartilage tissue of OA mice; (B) Western blot was used to detect the expression levels of ECM degradation and cartilage synthesis‐related proteins in the knee cartilage tissue of OA mice; (C) H&E and Safranin O–fast green staining of knee cartilage sections for histopathological assessment; (D) The OARSI scoring system was used to evaluate the severity of OA in mice; (E) ELISA was used to detect the levels of pro‐inflammatory cytokines IL‐8, IL‐6, and TNF‐α in knee cartilage tissue; (F) measurement of lactate content; (G) RT‐qPCR was used to detect glycolysis‐related genes, *n* = 6. Data are presented as mean ± SD. *p < 0.05, **p < 0.01.

**FIGURE 7:**
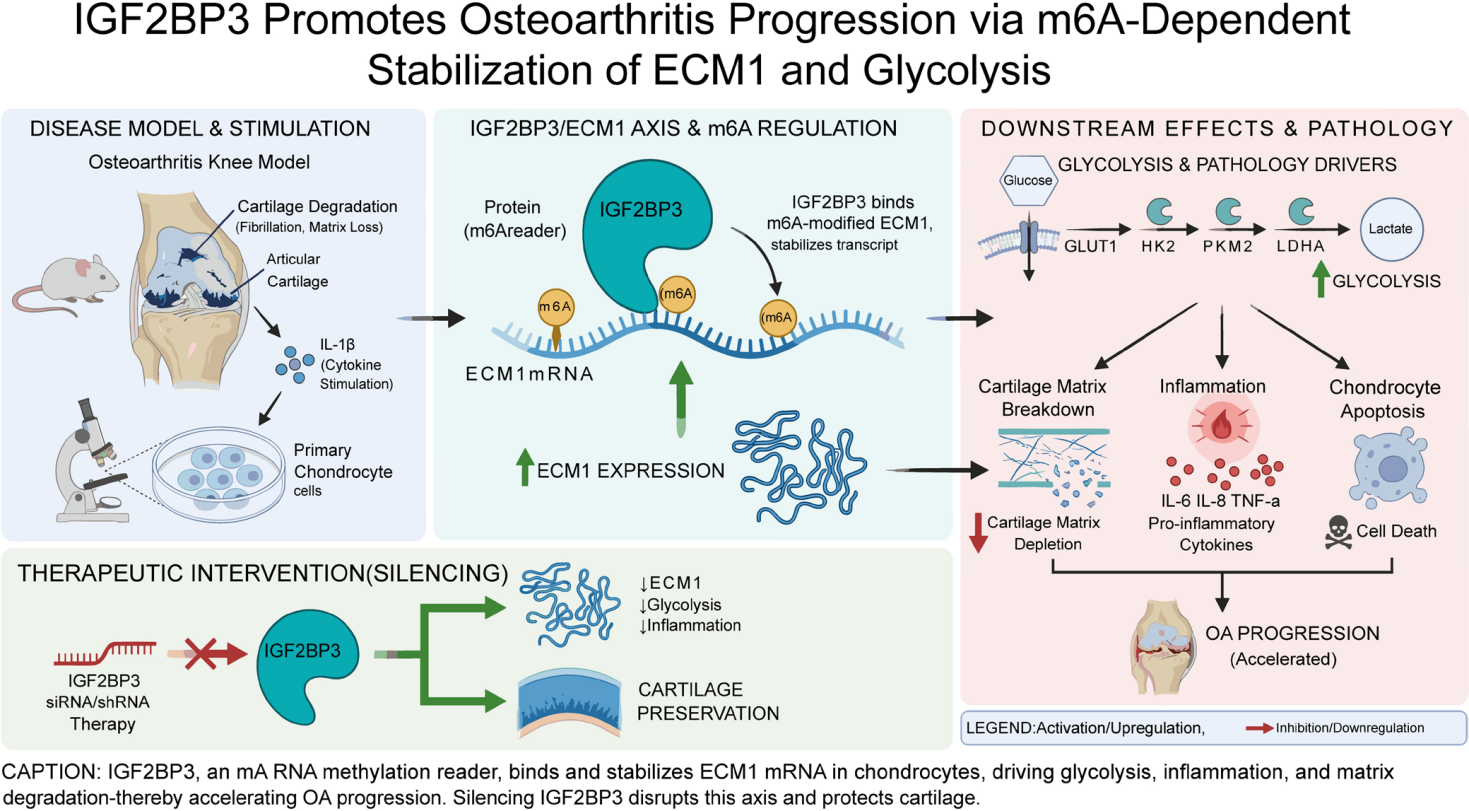
IGF2BP3 stabilizes ECM1 mRNA through an m^6^A‐dependent mechanism, thereby promoting glycolysis and accelerating OA progression. Knockdown of IGF2BP3 effectively inhibits OA progression.

The in vivo significance of IGF2BP3 in OA progression was further investigated using a DMM‐induced mouse model. Recombinant AAV encoding a short hairpin RNA targeting IGF2BP3 (AAV‐sh‐IGF2BP3) or a negative control (AAV‐NC) was administered via intra‐articular injection 1 week prior to DMM surgery. Immunohistochemical staining of knee cartilage sections confirmed effective knockdown of IGF2BP3 following AAV‐sh‐IGF2BP3 treatment, accompanied by a concurrent reduction in ECM1 expression (Figure 6A). Western blot analysis of cartilage tissues revealed that DMM surgery significantly upregulated the degradative enzymes MMP13 and ADAMTS5 while suppressing the anabolic markers collagen II and aggrecan compared with Sham controls. Notably, these OA‐associated alterations were markedly attenuated in mice receiving AAV‐sh‐IGF2BP3 (Figure 6B). Hematoxylin–eosin and safranin O‐fast green staining demonstrated severe cartilage destruction in the OA group, characterized by surface fibrillation, proteoglycan depletion, and reduced cellularity. In contrast, cartilage from IGF2BP3 knockdown mice exhibited preserved integrity with diminished histopathological features (Figure 6C). Consistently, OARSI scoring confirmed significantly lower cartilage damage in the AAV‐sh‐IGF2BP3‐treated group compared to untreated OA mice (Figure 6D). Biochemical analyses further revealed that DMM‐induced OA was associated with elevated levels of pro‐inflammatory cytokines (IL‐6, TNF‐α, IL‐8) and increased lactate concentration in cartilage tissue (Figure 6E,F). Moreover, the mRNA expression of glycolysis‐related genes GLUT1, LDHA, HK2, and PKM2 was upregulated in OA cartilage (Figure 6G). Importantly, targeted knockdown of IGF2BP3 effectively reversed these inflammatory and metabolic perturbations, resulting in reduced cytokine levels, lower lactate accumulation, and normalized expression of glycolytic enzymes (Figure 6E–G). Collectively, these findings demonstrate that IGF2BP3 knockdown exerts chondroprotective effects in vivo by attenuating cartilage degradation, suppressing inflammation, and inhibiting aberrant glycolytic activity, highlighting IGF2BP3 as a potential therapeutic target for OA (Figure 7).

## 4 Discussion

m^6^A modification has recently emerged as a key posttranscriptional regulator, governing mRNA stability, splicing, and translation efficiency [28]. Growing evidence underscores the significance of m^6^A in the pathogenesis of OA [29, 30]. The current investigation reveals that m^6^A methylation levels were markedly elevated in cartilage tissues from OA mice and in IL‐1β‐stimulated chondrocytes, a change potentially linked to the abundance of the m^6^A reader IGF2BP3. Previous work has demonstrated that IGF2BP3 stabilizes RRM2 transcripts in an m^6^A‐dependent manner to drive the malignant phenotype of hepatocellular carcinoma cells [31]. In line with this previous finding, the present data confirm that IGF2BP3 enhances the stability of ECM1 mRNA through m^6^A modification, thereby upregulating its expression and subsequently modulating aberrant glucose metabolism, processes that contribute to inflammatory responses and cartilage damage in OA.

As a key m^6^A reader, IGF2BP3 participates in regulating various inflammatory conditions, such as atopic dermatitis, airway inflammation, and infections, through its m^6^A‐binding activity [32, 33, 34]. Recent reports note that IGF2BP3 expression is increased in OA synovium, underscoring its connection with OA‐related inflammation [35]. Moreover, IGF2BP3‐mediated m^6^A modification facilitates proliferation, migration, invasion, and inflammatory cytokine secretion in rheumatoid arthritis fibroblast‐like synoviocytes; IGF2BP3 knockdown effectively attenuates disease severity [16]. Consistent with these observations, the present study detected significantly higher global m^6^A methylation levels along with elevated IGF2BP3 mRNA and protein expression in OA mouse cartilage relative to sham‐operated controls, and a pattern replicated in the cellular model. Importantly, knockdown of IGF2BP3 mitigated IL‐1β‐induced impairments in chondrocyte viability, and also suppressed apoptosis, inflammatory activation, and ECM degradation while also reducing overall m^6^A methylation. Furthermore, in vivo knockdown of IGF2BP3 ameliorated histopathological cartilage damage following DMM surgery and lowered OARSI scores. Collectively, these findings suggest that upregulated IGF2BP3 contributes to OA progression by mediating m^6^A‐dependent regulatory events.

Accumulating reports highlight the relevance of ECM1 in OA pathogenesis, and interventions targeting ECM1 have been proposed as a viable approach to curb disease advancement [23]. Correspondingly, this study observed substantial upregulation of ECM1 in both in vivo and in vitro OA models. To elucidate the mechanisms controlling its expression, and given the recognized role of IGF2BP3 in m^6^A methylation, bioinformatic analysis was performed, which predicted potential m^6^A modification sites within the 3′UTR of ECM1 mRNA. Subsequent experimental data indicated that under IL‐1β stimulation, elevated IGF2BP3 stabilizes ECM1 mRNA, leading to increased ECM1 protein expression. Consequently, overexpressing ECM1 counteracted the protective effects achieved by IGF2BP3 knockdown on OA progression.

Dysregulated glycolysis promotes inflammation and cartilage injury in OA [36]. Here, IL‐1β‐stimulated chondrocytes exhibited increased lactate production and glucose uptake, along with upregulated mRNA levels of key glycolytic enzymes. Analogous alterations were detected in cartilage from OA mice, implying an augmentation of glycolytic flux in cartilage under inflammatory stress [6, 37]. To further delineate the impact of IGF2BP3 on glycolysis, chondrocytes transfected with si‐IGF2BP3 were examined. IGF2BP3 knockdown markedly suppressed the glycolytic activity of chondrocytes exposed to IL‐1β, and consistent results were obtained from in vivo experiments. Nevertheless, certain limitations of this study should be acknowledged. Clinical validation is necessary to establish the correlation between IGF2BP3 expression and OA progression or patient prognosis. Furthermore, additional investigation is required to uncover other potential target genes or downstream signaling axes through which IGF2BP3 might regulate glycolysis in an m^6^A‐dependent manner involving ECM1.

In summary, this study demonstrates that IGF2BP3 and ECM1 are both upregulated in OA models. Knockdown of IGF2BP3 reduced m^6^A methylation on ECM1 mRNA, lowered glycolytic activity, and consequently reversed IL‐1β‐mediated suppression of chondrocyte proliferation, decreased apoptosis, attenuated inflammation and ECM degradation, and alleviated OA progression in mice. These findings provide novel targets and mechanistic insights for OA therapy.

## Funding

This work was supported by the National Natural Science Foundation of China (82072408).

## Conflicts of Interest

The authors declare no conflicts of interest.

## Data Availability

The data that support the findings of this study are available from the corresponding author upon reasonable request.
